# Composition of Metallic Elements and Size Distribution of Fine and Ultrafine Particles in a Steelmaking Factory

**DOI:** 10.3390/ijerph15061192

**Published:** 2018-06-07

**Authors:** Gabriele Marcias, Jacopo Fostinelli, Simona Catalani, Michele Uras, Andrea Maurizio Sanna, Giuseppe Avataneo, Giuseppe De Palma, Daniele Fabbri, Matteo Paganelli, Luigi Isaia Lecca, Giorgio Buonanno, Marcello Campagna

**Affiliations:** 1 Department of Medical Sciences and Public Health, University of Cagliari, 09042 Monserrato, Italy; michele_uras@hotmail.com (M.U.); andrea.sanna18@gmail.com (A.M.S.); avataneo@unica.it (G.A.); daniele.fabbri@hotmail.it (D.F.); isaialecca@gmail.com (L.I.L.); mam.campagna@gmail.com (M.C.); 2 Department of Medical and Surgical Specialties, Radiological Sciences, and Public Health, Section of Public Health and Human Sciences, University of Brescia, 25123 Brescia, Italy; j.fostinelli@unibs.it (J.F.); simona.catalani@unibs.it (S.C.); giuseppe.depalma@unibs.it (G.D.P.); m.paganelli002@unibs.it (M.P.); 3 Department of Civil and Mechanical Engineering, University of Cassino and Southern Lazio, I-03043 Cassino, Italy; giorgio.buonanno@uniparthenope.it; 4 International Laboratory for Air Quality and Health, Queensland University of Technology (QUT), Brisbane 4001, Australia; 5 Department of Engineering, University of Naples “Parthenope”, 80133 Naples, Italy

**Keywords:** ultrafine particles, metallic elements, occupational exposure

## Abstract

Background: The characteristics of aerosol, in particular particle size and chemical composition, can have an impact on human health. Particle size distribution and chemical composition is a necessary parameter in occupational exposure assessment conducted in order to understand possible health effects. The aim of this study was to characterize workplace airborne particulate matter in a metallurgical setting by synergistically using two different approaches; Methodology: Analysis of inhalable fraction concentrations through traditional sampling equipment and ultrafine particles (UFP) concentrations and size distribution was conducted by an Electric Low-Pressure Impactor (ELPI+™). The determination of metallic elements (ME) in particles was carried out by inductively coupled plasma mass spectrometry; Results: Inhalable fraction and ME concentrations were below the limits set by Italian legislation and the American Conference of Governmental Industrial Hygienists (ACGIH, 2017). The median of UFP was between 4.00 × 10^4^ and 2.92 × 10^5^ particles/cm^3^. ME concentrations determined in the particles collected by ELPI show differences in size range distribution; Conclusions: The adopted synergistic approach enabled a qualitative and quantitative assessment of the particles in steelmaking factories. The results could lead to a better knowledge of occupational exposure characterization, in turn affording a better understanding of occupational health issues due to metal fumes exposure.

## 1. Introduction

The issue of particles exposure in metallurgical industries is of particular concern because it can have a negative impact on the workers’ health [1,2,3,4,5,6].

Although traditionally the evaluation of exposure was focused on the mass concentration of inhaled dust, the characteristics of aerosol, in particular particle size and chemical composition, can have an impact on human health. In recent years, some studies have suggested that adverse pulmonary and cardiovascular health effects are associated with ultrafine particles (UFP) [7,8,9,10,11], which are defined as particles not deliberately produced, and with a diameter <100 nm. Some toxicological and epidemiological studies have shown that UFP are more toxic than large particles [12,13] because they are more reactive and behave differently in the respiratory system, where they can deposit in the alveoli region and interact with epithelial cells [14,15,16]. Other authors have highlighted that UFP can cause oxidative stress, which may play a major role in the development of adverse health effects [11,17].

Industrial settings, such as foundries of ferrous and non-ferrous materials, can represent potential relevant sources of UFP exposure both for employed workers and for populations living near to industrial plants [18,19,20,21], compared to outdoor ambient levels measured in urban and rural areas [22,23]. The airborne particles emitted from industrial plants, such as steel foundries, can be made up of a mix of toxic pollutants, such as volatile organic compounds and heavy and transition metals, that could be responsible for adverse health effects [24,25,26,27,28].

Some studies described different patterns in terms of the distribution of metallic elements (ME) in different granulometric fractions, in particular UFP, when collecting samples in the proximity of metallurgical settings [29,30], suggesting possible negative health effects in exposed subjects. However, it is still not clear if and how the chemical composition of UFP can contribute to or produce adverse health effects compared to the physical effect of UFP itself [31,32,33].

Understanding particle size distribution not only in terms of mass, and its chemical composition is a necessary parameter in environmental and occupational exposure assessment for understanding the possible effects to health [26,34,35,36].

In this sense, a proper and in-depth exposure characterization could help to design epidemiological studies in order to better understand the health effects of different components within different particulate matters.

There are several measurement strategies which can be used to assess and characterize the airborne particulate matter in an industrial plant [26,37]. Recently, the Electric Low-Pressure Impactor (ELPI+™, Dekati, Tampere, Finland) has been successfully applied to assess the aerosol distribution and total number concentration evolution in metalworking industries [38,39,40,41]. All these studies provide a qualitative analysis of the chemical composition of fine particles and UFP by means of electronic scan microscopy of the collected aerosol with poor data on the quantitative distribution of ME with higher toxicological relevance, such as arsenic, chromium, cadmium, and manganese, in the particulate spectrum.

Despite the great potential use of ELPI for the assessment of airborne particulate, few studies have been carried out that synergistically use the results obtained by ELPI with those of traditional sampling techniques for airborne workplace pollutants.

Thus, the aim of this study was to characterize workplace airborne particulate matter in a metallurgical setting by synergistically using two different approaches:

(1) the sampling of inhalable fraction, the measurement of their concentration, and their chemical characterization in terms of ME;
(2) the measurement of number concentrations and particle size distributions carried out by ELPI.

A further purpose was to test the possibility of carrying out a multi-elemental analysis through inductively coupled plasma mass spectrometry (ICP-MS) on the particulate collected by ELPI.

## 2. Materials and Methods

### 2.1. Sampling Site

The steel foundry plant specializes in steels for the construction industry; it makes use of electric arc furnace (EAF) “Mini Mills” for the processing of molten steel in the ladle and subsequent casting in a continuous plant. The facility produces steel billets destined to feed in real time the rolling plant. The raw material is composed of iron scrap.

The melting is essentially done by a three-phase furnace equipped with three electrodes in graphite; the arc strikes between the ends of each of the three electrodes and the metal charge. The electric power is converted into heat, up to a temperature of 2000 °C. 

The plant produces concrete reinforcing bars of 12 mm to 40 mm in diameter and 5 m to 26 m in length.

The different qualities of steel are obtained by following essentially the same production process.

Through refining in the ladle furnace, one can obtain specific compositions and control the quality of the steel (the exact composition of the alloy is proprietary information).

The sampling sites in this study are the ladle furnace, the continuous casting area, and the outdoor plant near to the electric arc furnace.

### 2.2. Experimental Design

**Figure 1:**
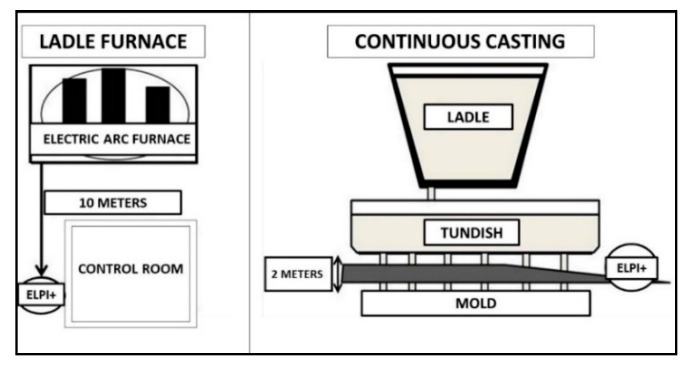
Schematic diagrams of the indoor sampling sites in the steel foundry: Ladle furnace sampling site (**left**) and continuous casting sampling site (**right**).

**Figure 2:**
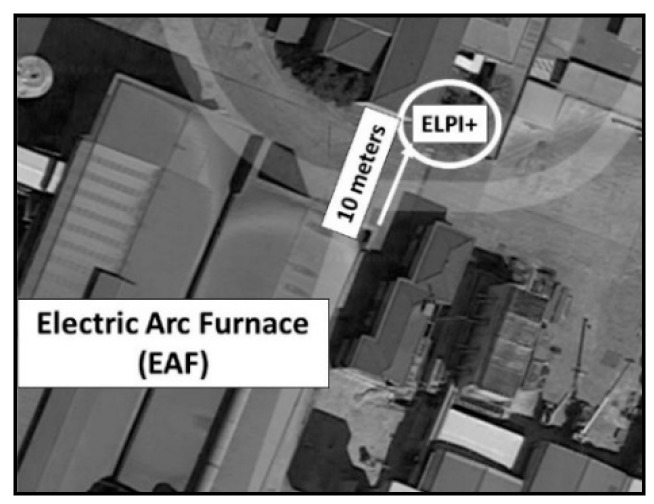
Outdoor sampling site: Sampling site outside of the plant near the electric arc furnace.

Environmental samplings were carried out in three different areas of the factory during standard working conditions. Two indoor samplings were conducted, one at a 10 m distance from the ladle furnace, and the other next to the continuous casting area at a distance of 2 m from the tundish (Figure 1). Afterwards, one sampling was conducted outside the plant at a distance of 10 m from the electric arc furnace to investigate the presence of particulate matter in the outdoor environment (Figure 2). The main road, which was the closest to the outdoor sampling site, was approximately 500 m away (A4 highway).

The duration of the samplings lasted approximately 1 h and 30 min in each location. As a main purpose, the samplings were conducted by synergistically using two different approaches; at the same time, powder inhalable fraction concentrations were measured through traditional sampling equipment, and particle concentrations and size distribution were determined by ELPI. Afterwards, chemical characterization focused on ME was conducted on the collected materials from both samples. Considering previous experiences [42] describing very small masses of UFP, the chemical characterization on the ELPI-sampled material was carried out for some stages, although the ideal duration of sampling to obtain sufficient mass was not known.

### 2.3. Sampling Equipment

The measurement of inhalable fraction concentration was performed in accordance with the UNI EN 481 Italian standard method [43], using stationary samplers at a constant flow of 2 L/min. The particulate was collected through an Institute of Occupational Medicine (IOM) sampler (IOM Sampler, SKC Inc., Eighty Four, PA, USA) and mixed cellulose esters membrane filters (diameter: 25 mm; porosity 0.8 µm), according to Italian standards [44]. The pumps were placed in the center of the sampling areas and the IOM samplers were positioned approximately 1.8 m off the ground.

The sampling time of the inhalable fraction ranged from three to six hours in each location. During the sampling, the aspirated air flow was conducted at the ladle furnace (770 L), continuous casting (420 L), and outside of the plant near the electric arc furnace (420 L). It was not possible for the samplings to be carried out with the same sampling times, for logistical reasons. Particle concentrations and size distribution monitoring were carried out with the ELPI+™ (Dekati^®^, Tampere, Finland). Detailed descriptions of the ELPI+™ function and its principles of operation are given in the literature [45]. The ELPI+™ is connected to a vacuum pump with a flow rate of 0.6 m³/h and a pressure of 40 mbar measured at the final stage of the impactor. Particles are collected at the different impactor stages depending on their aerodynamic diameter; the electric charge of the particles, instantaneously measured by the electrometers, is directly proportional to the particle number. Finally, the instrument provides an estimate of the area/mass/volume of the particulate. Particle size distribution and concentration are measured in the size range between 0.006–10 μm with a 10 s sampling rate. The ultrafine particle number concentration was calculated as the sum of the particles within the 6–94 nm size range (50% cut-off size diameter, D50%), with a geometric mean aerodynamic diameter (Di) range of 10 to 72 nm. The assumed density values were 1 g/cm³, which is in line with previous studies [42]. The number-to-mass conversion was performed using the same assumed density, utilizing the filter functions in the ELPI_VI_ software [46]. The particles were collected in the ELPI on polycarbonate foil substrates from the fifth to fourteenth stage.

### 2.4. Chemical Characterization

Determination of ME (Al, As, Ba, Be, Cd, Co, Cr, Cu, Fe, Hg, Mn, Mo, Ni, Pb, Sb, Sn, Sr, Zn) was carried out in the collected particles from both samples. One sample for each fraction was analyzed. These particle samples were analyzed by ICP-MS analysis on a Perkin Elmer ELAN DRC II instrument (Perkin Elmer Sciex, Woodbridge, ON, Canada) equipped with dynamic cell reaction (DRC) to analyze chromium and iron.

The mixed cellulose esters membrane filters and the polycarbonate foil substrates were extracted overnight in a nitric acid (HNO_3_) ACS Reagent (Purity ≥ 90.0%; Sigma, Milan, Italy) 70% (*v*/*v*), and the extracted samples were diluted into Ultrapure deionized water (Tracepure^®^ water for inorganic analysis, Merck, Rome, Italy).

Prepared samples underwent inductively coupled plasma mass spectrometry (ICP-MS) analysis by using the analytical technique total quant with external calibration. For each sample, two runs were performed (two replicates each), one with dynamic reaction cell (DRC) and one without DRC. The instrumental conditions for multi-elemental analysis by ICP-MS and specific technical details have been reported in the previous study [47]. The reagent blank was made from blank membranes, acid, and deionized water used for the sampled membranes. The instrument was calibrated using standard solution at a concentration of 10 µg/L (Multielement ICP-MS Calibration Standard 3, Matrix per Volume: 5% HNO_3_ per 100 mL, Perkin Elmer Plus). The limits of detection (LOD) were determined on the basis of three standard deviations (SD) of the background signal; LOD ranged from 0.0001 µg to 0.0006 µg and the coefficient of variation ranged from 6.5% to 9%.

The accuracy of the method was determined on the basis of the mean values obtained on certified reference materials submitted to the same treatment as the samples (Trace Elements in water NIST 1640). Using this validated method, the laboratory obtained successful results in external quality assessment schemes organized by the Institute for Occupational, Environmental, and Social Medicine of the University of Erlangen, Germany (G-EQUAS program)

The limit of detection laboratories were accredited (to ISO 9001:2000 no. 9122 SP 16).

## 3. Results

### 3.1. Determination and Chemical Characterization of Inhalable Fraction

Airborne inhalable fraction concentrations and concentrations of ME are reported in Table 1. Regarding dust concentrations, higher levels were found in the continuous casting sampling area, while lower levels were found in the outdoor sampling area.

Among ME, all concentrations were higher in the continuous casting sampling area, except for Mn, in which levels were higher in the ladle furnace sampling area. Be was below the limit of detection in all samples. All levels measured outdoors were found to be lower compared to the results of the indoor sampling. Both inhalable fraction and ME concentrations were below the limits set by Italian legislation and the American Conference of Governmental Industrial Hygienists (ACGIH) [48,49].

### 3.2. Particle Number Concentrations

**Figure 3:**
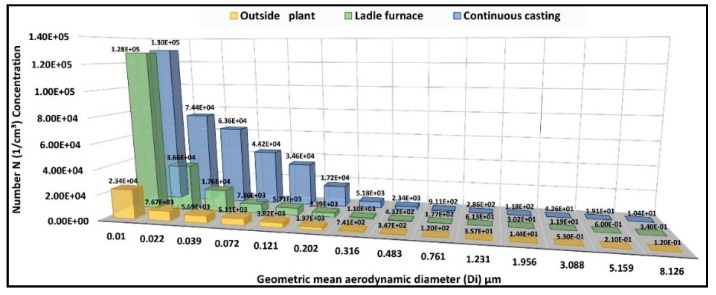
Mean values of the particle number concentrations (particles/cm^3^) in 14 fractions (reported as geometric mean aerodynamic diameter (Di µm)) measured by Electric Low-Pressure Impactor (ELPI).

In general, for most of the fractions, higher values were obtained from the sampling in the continuous casting area, showing values up to an order of magnitude higher for some fractions (Figure 3). The only exceptions are related to the 0.010 μm size, in which the particle number concentration was comparable to ladle furnace sampling. The concentrations measured outside the production plant were lower than those measured inside in all the sampled fractions.

**Figure 4:**
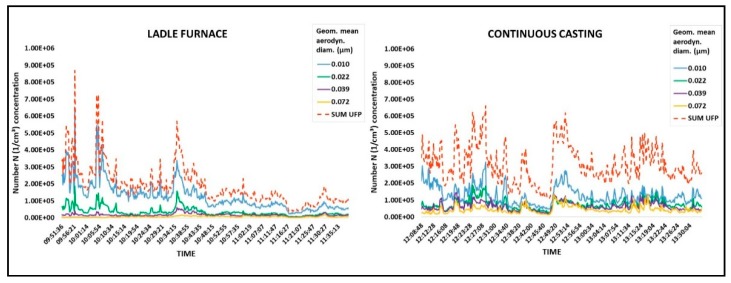
Time variation of ultrafine particle (UFP) number concentrations (part/cm^3^) by size measured by ELPI next to ladle furnace and continuous casting. *x*-axis represents sampling (hh:mm:ss) and *y*-axis represents UFP number concentration (1/cm^3^).

Figure 4 shows the time variations of ultrafine particle (D50% 6–94 nm; Di 10–72 nm) number levels over the sampling period next to the ladle furnace and continuous casting. The measurement results obtained next to the ladle furnace and continuous casting for UFP number concentrations were 3.90 × 10^4^ to 8.70 × 10^5^ particles/cm^3^ (median 1.64 × 10^5^ particles/cm^3^) and 1.06 × 10^5^ to 6.61 × 10^5^ particles/cm^3^ (median 2.92 × 10^5^ particles/cm^3^), respectively.

**Figure 5:**
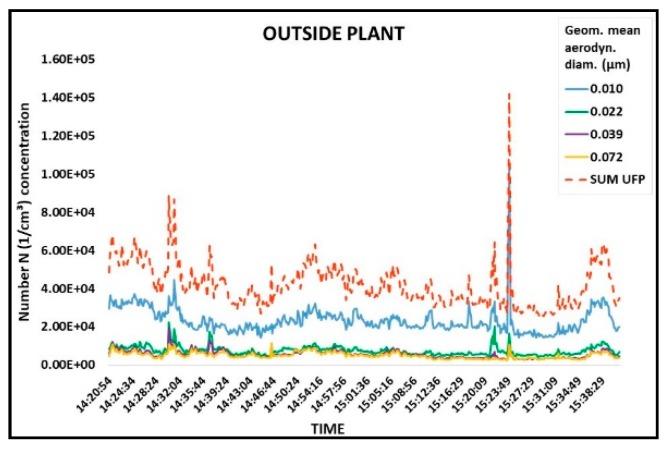
Time variation of UFP number concentrations (part/cm^3^) by size measured with ELPI outside of the plant at a distance of 10 m from the electric arc furnace in the steel foundry. The *x*-axis represents sampling (hh:mm:ss) and the *y*-axis represents UFP number concentration (1/cm^3^).

Figure 5 shows the time variations of ultrafine particles (D50% 6–94 nm; Di 10–72 nm) number levels over the sampling period outside the plant. The ultrafine particles number levels in the environment outside the plant were 2.57 × 10^4^ to 1.42 × 10^5^ particles/cm^3^ (median 4.00 × 10^4^ particles/cm^3^).

### 3.3. Particle Number and Mass Size Distribution

**Figure 6:**
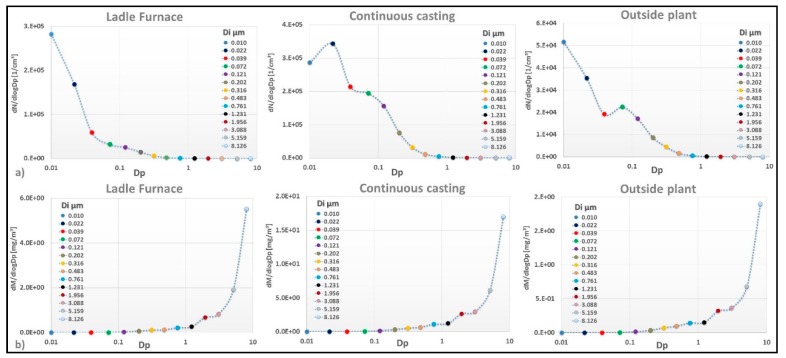
Particle number (**a**,**b**) mass distribution measured with the ELPI for locations (ladle furnace, continuous casting, and outside the plant).

In general, the particles composing aerosol were very small, with 83–95% of the particle number distribution measured using the ELPI < 0.100 µm. Figure 6a,b provides number and mass distribution of the particles (Di size range 0.010 μm–8.126 μm) measured in the ladle furnace, continuous casting, and outside the plant, respectively. Measurements obtained near the continuous casting area had elevated particle number concentrations in the size range 0.022 µm; a higher presence of small particles in the size range between 0.072 and 0.316 μm was also shown.

On the other hand, the particle number by size distribution for the fume measured next to the ladle furnace showed a peak at 0.010 µm, with 63% of the total particle number. Sampling outside showed that most particles were in the size range 0.010 µm, with an additional possible peak at 0.072 µm.

Mass distributions exhibited one possible main mode at around 8.126 µm (Figure 6b).

### 3.4. Chemical Characterization of Particles Collected by ELPI+

**Figure 7:**
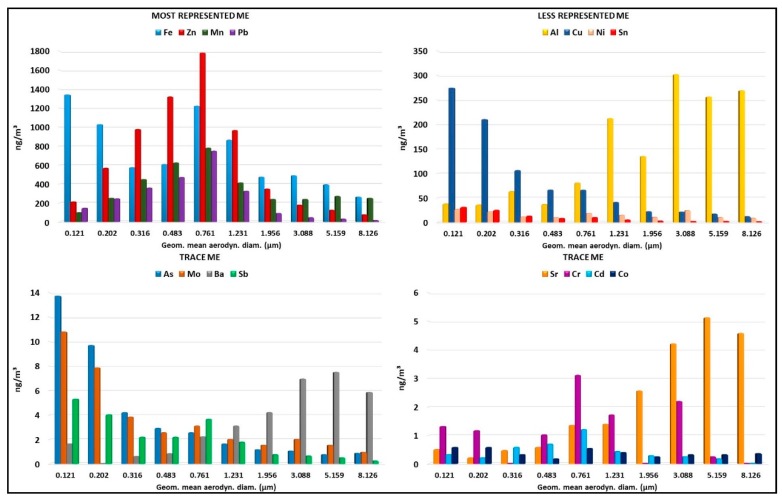
Mass concentration (ng/m^3^) of ME determined in the different particle size ranges collected by ELPI. The *x*-axis represents the geometric mean aerodynamic diameter (Di µm) and the *y*-axis represents the mass concentration of ME (ng/m^3^).

Figure 7 shows the mass concentration distribution of the elements determined in the different particle size ranges collected by ELPI (Supplementary Materials).

The most represented ME were Fe 32%, Zn 29%, Mn 16%, and Pb 11%, while less represented ME were Al 6%, Cu 4%, Ni 1%, and Sn 0.5% of the total distribution in the particulate collected by ELPI. The trace ME were As, Mo, Ba, Sb, Sr, Cr, Cd, and Co. The Hg and Be values were below detection limits in all fractions collected by ELPI (data not shown).

The most represented elements (Fe, Zn, Mn, and Pb) increase their contribution to the total amount in fine particles. In particular, 62–77% of the total amount of Zn, Mn, and Pb was detected in the size range between 0.316 µm and 1.231 µm. Iron shows a similar distribution, with 77% of the total in the size range between 0.121 µm and 1.231 µm. The mass distribution for these elements shows one peak at 0.761 µm size fraction, except Fe, which shows an additional peak at 0.121 µm; Sb shows a similar pattern to Fe. 69% of the total amount of Cd was detected in the size range between 0.316 µm and 1.231 µm, and 28.6% of the total amount was detected in the 0.761 µm size fraction.

On the other hand, the minor elements show a different distribution in the size range compared to the major elements. In particular, Al shows a similar distribution to Ba and Sr: These elements increase their contribution to the total amount with the increasing of particle size.

A Almost 80% of the total amount of Al, Ba, and Sr was detected in the size range between 1.231 µm and 8.126 µm. Cu and Sn elements show a clear trend, and their distribution is quite similar to As and Mo elements. Cu, Sn, As, and Mo increase their contribution to the total amount with a decrease of particle size to below 0.3 µm. About 30% of these elements were found in the fraction of 0.121 µm.

Finally, some toxic elements, such as Ni, Co, and Cr, do not show a clear trend. Ni and Co show almost uniform distribution for all size ranges, whereas Cr shows high variation in the fraction size ranging from concentrations lower than the detection limit value (0.316 µm, 1.956 and 8.126) to about 30% (0.761 µm) of the total element in a single fraction. However, Ni and Co account for more than 50% in small particles <0.8 µm.

## 4. Discussion

The adopted environmental monitoring strategy has allowed some new insights into the characterization of particulate emissions in workplaces of particular concern for occupational and environmental health, such as steelmaking factories. In particular, to the best of our knowledge, no studies have reported the results of the synergistic sampling strategy performed in this study. The level of inhalable fraction concentration (Table 1) and particle number concentration are in agreement with each other. The highest particulate levels were measured next to the continuous casting area in terms of mass concentration (inhalable fraction, mg/m^3^) and in terms of particle number concentration (median of UFP part/cm^3^). Although the measurements carried out are not sufficient to assess the airborne particulate matter in the whole plant, the results are comparable to other studies, which have shown that melting and pouring operations can generate high concentrations of fine and ultrafine particles compared to outdoor particles levels [50]. Furthermore, the high variability of the concentration in the number of UFP observed next to the ladle furnace and continuous casting areas (Figure 4), as suggested by other studies, could be due to different steps of the production cycle [28,41,51]. Further research is needed to examine in depth the time variation of UFP number concentrations inside the steelmaking factory in order to provide useful information to identify possible prevention measures aimed at containing emission peaks or providing protection strategies [41].

Higher mean values of the UFP near the continuous casting area, compared with the ladle furnace, are in agreement with a previous investigation [52], which reported that a pouring operation contributed to the highest concentration emission of aerosol. The UFP concentrations measured in the indoor steel foundry were similar to those measured by Evans et al. (2007) [50] in an automotive grey iron foundry, and Cheng et al. (2008) [51] in an iron foundry, with 7.0 × 10^4^ to 2.39 × 10^5^ particles/cm^3^ and 2.07 × 10^4^ to 2.82 × 10^5^ particles/cm^3^, respectively.

Despite the proximity to the plant, overall, the mean values of particulate number measured outside the plant in the different size fractions were lower than the mean values measured inside the factory. In particular, the majority of the outdoor mean values were up to an order of magnitude lower than the mean values measured next to the continuous casting area (Figure 3).

The UFP number in the environment outside the steel factory (median 4.00 × 10^4^ particles/cm^3^) was comparable to that reported in two previous studies of similar areas outside plants [50,51], which measured levels ranging from 3.30 to 3.69 × 10^4^ and 1.26–1.89 × 10^4^, respectively.

The particle number by size distribution for the fume generated at the casting process (Figure 6a) appeared to be characterized by a main mode around 0.022 μm; a higher presence of the small particles in the size range between 0.072 and 0.316 μm was also shown. Chang et al. (2005) [52] measured three modes in the particle number distribution during the casting process in the fraction 0.030–0.060 µm, 0.170–0.650 µm, and 0.030–0.260 µm; also, Evans et al. (2007) [50] measured the smallest and dominant mode, <0.023 µm, which was generated at the rotopour process, and observed other additional modes at 0.04 µm and 0.2 µm.

As suggested by previous studies, the largest particle size of the emission fumes of the casting process could depend on a vapor species available for condensation and coagulation [52]. In contrast, the smallest particles measured next to the ladle furnace (diameter 0.010 µm) fumes were likely composed of freshly nucleated particles. Mass distribution (Figure 6b) is similar in all measures, as more than 90% of mass is in the particles >1 μm, and ultrafine mass is <1% of the total. The sampling strategy allows for the determination of a large number of ME, both on the inhalable fraction and ELPI, taking advantage of the analysis carried out with ICP-MS.

Airborne concentrations of ME in the inhalable fraction (D50% 100 µm) (Table 1) were below the threshold limit values (TLV-TWA) proposed by ACGIH for 2017 [49]. Regarding the particulate collected by ELPI, concentrations were assessed for most ME, carrying out for the first time, to the best of our knowledge, a qualitative and quantitative chemical assessment of fine particles and UFP in this workplace environment; however, the number of samples and sampling time were limited.

In the particulate matter collected by ELPI, the most represented ME were Fe, Zn, and Mn. This distribution reflects the proportions and the chemical composition expected in steel production. 

Although further studies are needed to confirm the repeatability of the results, the concentration of ME, determined in the particulate collected by ELPI, shows differences in the particles’ size distribution (Figure 7). As evidenced by other studies, the different proportions of the metal elements in the particle size fractions may be due to different chemical-physical characteristics of the ME, which may be related to the boiling point temperature of the different metals [53].

It is known that ME carcinogens have in common the main route of exposure, which is inhalation, and the main target organs of the respiratory tract [54]. Among the supposed action mechanisms that make their carcinogenic action plausible, some studies have reported their ability to penetrate the cell and interact with target sites, such as DNA. ME can generate reactive oxygen species (ROS) and other intermediates that can cause direct DNA damage by interacting with enzymes involved in its repair, as well as with regulators of cell proliferation [27,55]. Some toxicological studies have shown that the nanoparticles of metallic oxides produced in high temperature processes are absorbed by cells; they then release metal ions inside [56,57,58]. Furthermore, a recent study has shown that UFP generated by welding activities containing toxic metals have a greater ability to induce reactive oxygen species (ROS) than larger particles, regardless of mass concentration [59]. A recent study examined the relation between acute changes in cardiovascular and respiratory function and metals contained in particulate matter (PM 2.5) produced by steel plants [26]. The authors observed that several metals contained in the fine particles were associated with acute changes in cardiovascular and respiratory physiology. They questioned whether PM-associated metals might play a role in causing health effects. A comprehensive exposure characterization could help to better understand the toxicological mechanisms involved in the pathogenesis of adverse health effects in exposed subjects. In this sense, the adopted synergistic approach allowed the characterization of different distributions of ME in the particle size fractions, with a predominance of some toxic metals, such as arsenic, lead, nickel, and chromium, in the smaller fractions (fine and ultrafine). This methodological approach seems to represent a useful tool for better understanding the characterization of occupational exposure in steel factories.

Further studies are warranted to extend the sampling strategy by assessing other sampling sites and pollutants other than metallic elements, inside and outside the ferroalloy plant, to better characterize workers’ personal exposure.

## 5. Conclusions

The characterization of airborne particulate matter in a steelmaking factory has been performed. Indoor levels of ultrafine particle number concentrations and inhalable fraction concentrations were found to be higher than those in the outdoor environment. These data confirm previous findings described in similar industrial settings. The adopted synergistic approach enabled a qualitative and quantitative assessment of the particles in the work environment and to observe a different distribution of ME in different particulate fractions. These results confirm that the ELPI, together with other instruments, can be used to improve knowledge of the characterization of airborne particulate matter in the metallurgical setting. This could help to develop job-based exposure matrices useful for designing epidemiological studies in order to improve knowledge of the health effects of different components within different particulate matters.

## Figures and Tables

**Table 1:** Concentrations of the inhalable fraction (mg/m^3^) and chemical composition of the particulate collected analyzed by inductively coupled plasma mass spectrometry (ICP-MS), expressed as concentrations (µg/m^3^) of the metallic elements (ME) obtained from sampling in the ladle furnace, continuous casting, and outside the plant.

Sampling Sites		Ladle Furnace		Continuous Casting		Outside Plant	
**Inhalable Fraction Concentration**		0.81		1.19		0.4	
**Metallic Elements**	**Sampling Sites**			**Metallic Elements**	**Sampling Sites**		
	**Ladle Furnace**	**Continuous Casting**	**Outside Plant**		**Ladle Furnace**	**Continuous Casting**	**Outside Plant**
**Al**	6.243	7.223	2.88	**Hg**	0.0012	0.0018	<0.0005 ^1^
**As**	0.0565	0.2856	0.0035	**Mn**	41.016	15.16	0.6973
**Ba**	0.1947	0.3034	0.2142	**Mo**	0.0487	0.2737	0.0212
**Be**	<0.0006 ^1^	<0.0006 ^1^	<0.0006 ^1^	**Ni**	0.2467	0.833	0.1107
**Cd**	0.0043	0.0134	0.0024	**Pb**	3.122	5.093	0.3094
**Co**	0.0143	0.0274	0.0041	**Sb**	0.0208	0.1095	0.0093
**Cr**	0.095	0.158	0.044	**Sn**	0.1181	0.595	0.0357
**Cu**	0.7983	4.272	0.1785	**Sr**	0.1188	0.1737	0.0821
**Fe**	2.132	40.579	0.0405	**Zn**	6.827	8.794	1.82

^1^ Below the LOD (Limit of detection, µg).

## Supplementary Materials

The following are available online at http://www.mdpi.com/1660-4601/15/6/1192/s1. Original data of particles concentration, distribution and chemical characterization of particles collected by ELPI+ are reported in the Supplementary Materials “*Chemical characterization of particles collected by ELPI; data of sampling continuous casting.xlsm; data of sampling ladle furnace.xlsm; data of sampling outside plant.xlsm*”.
