# Supplementary material for: Composition of Metallic Elements and Size Distribution of Fine and Ultrafine Particles in a Steelmaking Factory
Source: Int J Environ Res Public Health. 2018 Jun 7;15(6):1192. doi: 10.3390/ijerph15061192 (PMC6025616; doi:10.3390/ijerph15061192)

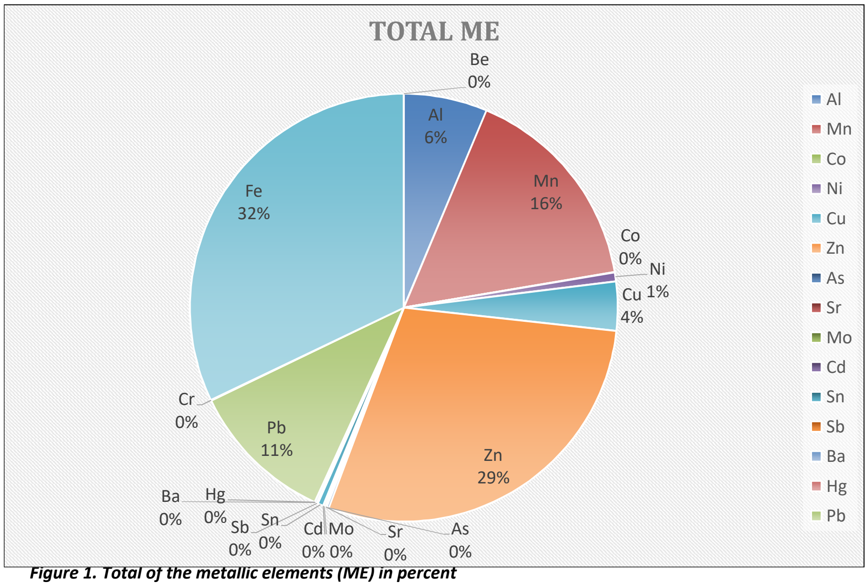

Figure 1. Total of the metallic elements (ME) in percent

Table 1. Concentration of ME (ng/m³) by size fraction

Di (µm) stage ELPI+	METALLIC ELEMENTS ng/m³			
Most represent	Fe	Zn	Mn	Pb
0.121	1345.59	213.24	99.63	147.06
0.202	1031.25	569.85	251.84	246.32
0.316	577.21	979.78	450.37	360.29
0.483	610.29	1323.53	626.84	472.43
0.761	1226.10	1786.76	783.09	751.84
1.231	867.65	968.75	415.44	327.21
1.956	476.10	349.26	240.81	91.91
3.088	490.81	180.15	240.81	47.06
5.159	395.22	125.00	272.06	33.09
8.126	264.71	77.21	251.84	18.38

Di (µm) stage ELPI+	METALLIC ELEMENTS ng/m³			
Less represent	Al	Cu	Ni	Sn
0.121	38.24	275.74	27.02	31.25
0.202	36.03	211.40	21.88	25.00
0.316	63.60	106.62	11.40	12.87
0.483	36.76	66.54	9.93	8.27
0.761	80.88	66.18	18.38	10.11
1.231	213.24	41.18	14.71	5.15
1.956	135.29	22.06	10.66	2.76
3.088	303.31	21.32	24.26	2.10
5.159	257.35	17.28	10.11	2.21
8.126	270.22	12.13	8.64	1.36

Di (µm) stage ELPI+	METALLIC ELEMENTS ng/m³							
Trace ME	As	Mo	Ba	Sb	Sr	Cr	Cd	Co
0.121	13.79	10.85	1.65	5.33	0.51	1.32	0.33	0.59
0.202	9.74	7.90	0.02	4.04	0.22	1.18	0.22	0.59
0.316	4.23	3.86	0.63	2.21	0.48	0.02	0.59	0.33
0.483	2.94	2.57	0.85	2.21	0.59	1.03	0.70	0.18
0.761	2.57	3.13	2.24	3.68	1.36	3.13	1.21	0.55
1.231	1.65	2.02	3.13	1.80	1.40	1.73	0.44	0.40
1.956	1.18	1.54	4.23	0.77	2.57	0.02	0.29	0.26
3.088	1.07	2.02	6.99	0.66	4.23	2.21	0.26	0.33
5.159	0.77	1.54	7.54	0.51	5.15	0.26	0.18	0.33
8.126	0.88	0.96	5.88	0.26	4.60	0.02	0.02	0.37

Table 2. Concentration of ME (ng) by size fraction

Di (µm) stage ELPI+	METALLIC ELEMENTS ng																
	Al	As	Ba	Be	Cd	Co	Cr	Cu	Fe	Hg	Mn	Mo	Ni	Pb	Sb	Sn	Sr	Zn
0.121	104	37.5	4.5	<0,6	0.9	1.6	3.6	750	3660	<0,5	271	29.5	73.5	400	14.5	85	1.4	580
0.202	98	26.5	<0,1	<0,6	0.6	1.6	3.2	575	2805	<0,5	685	21.5	59.5	670	11	68	0.6	1550
0.316	173	11.5	1.7	<0,6	1.6	0.9	<0,1	290	1570	<0,5	1225	10.5	31	980	6	35	1.3	2665
0.483	100	8	2.3	<0,6	1.9	0.5	2.8	181	1660	<0,5	1705	7	27	1285	6	22.5	1.6	3600
0.761	220	7	6.1	<0,6	3.3	1.5	8.5	180	3335	<0,5	2130	8.5	50	2045	10	27.5	3.7	4860
1.231	580	4.5	8.5	<0,6	1.2	1.1	4.7	112	2360	<0,5	1130	5.5	40	890	4.9	14	3.8	2635
1.956	368	3.2	11.5	<0,6	0.8	0.7	<0,1	60	1295	<0,5	655	4.2	29	250	2.1	7.5	7	950
3.088	825	2.9	19	<0,6	0.7	0.9	6	58	1335	<0,5	655	5.5	66	128	1.8	5.7	11.5	490
5.159	700	2.1	20.5	<0,6	0.5	0.9	0.7	47	1075	<0,5	740	4.2	27.5	90	1.4	6	14	340
8.126	735	2.4	16	<0,6	<0,1	1	<0,1	33	720	<0,5	685	2.6	23.5	50	0.7	3.7	12.5	210

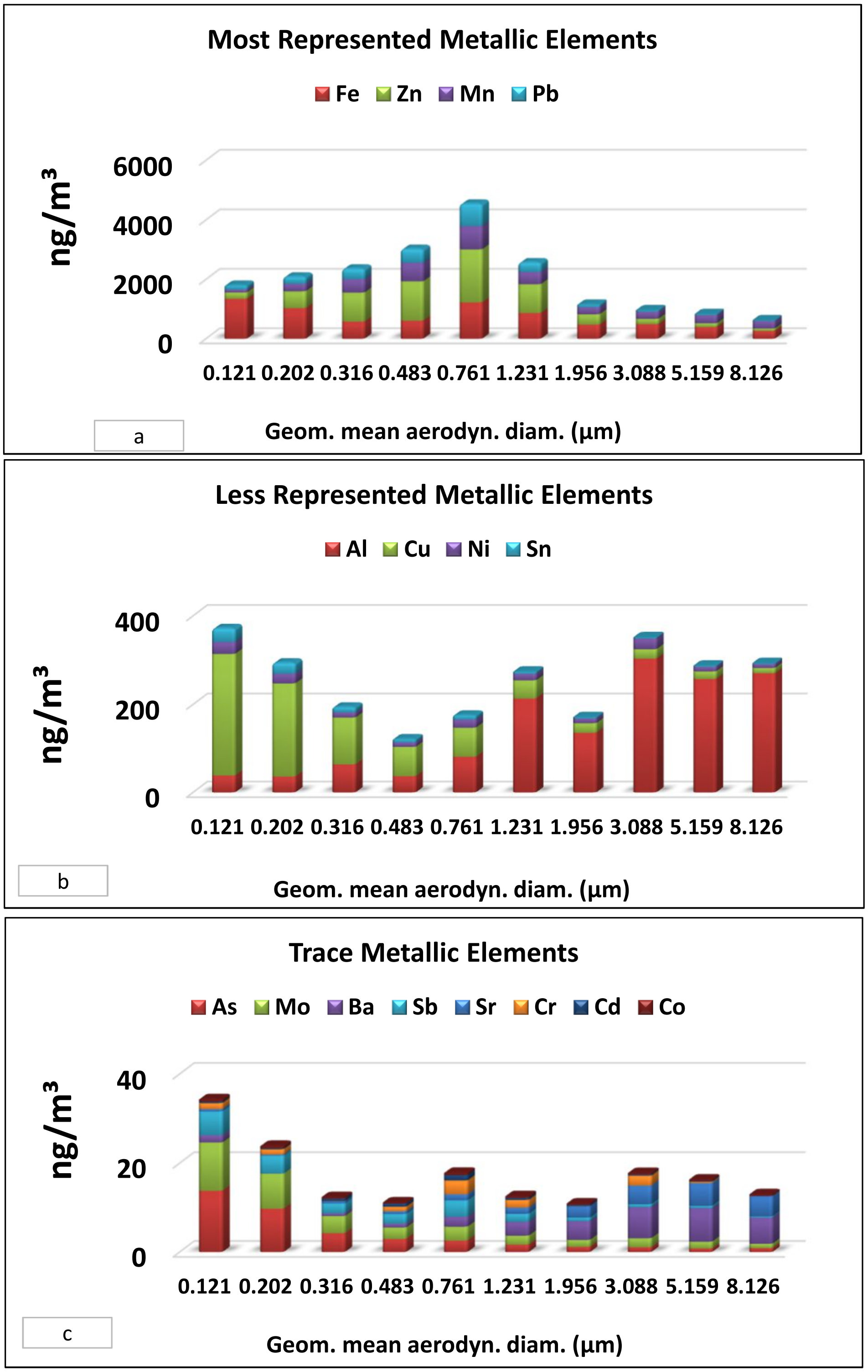

Figure 2. Distribution of most represented ME (a), less represented ME (b) and trace ME (c)

METALLIC ELEMENTS BY SIZE FRACTION

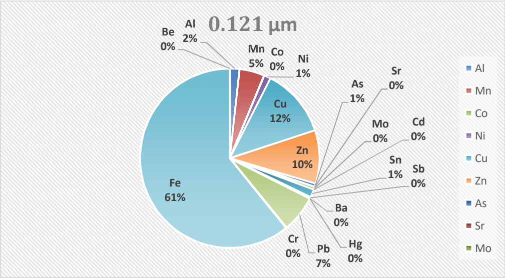

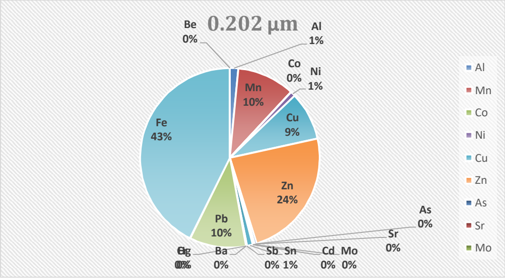

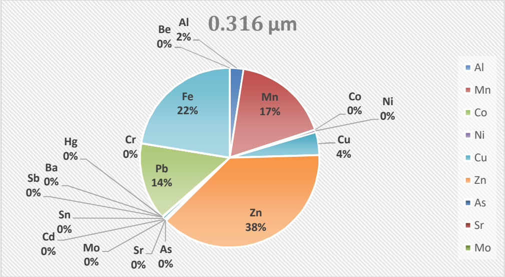

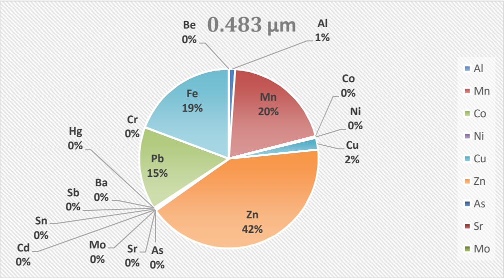

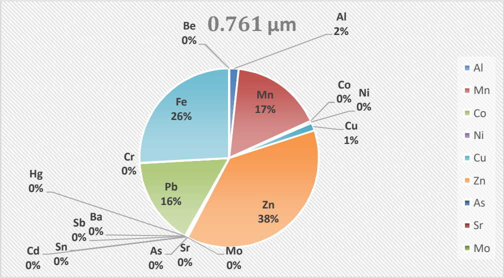

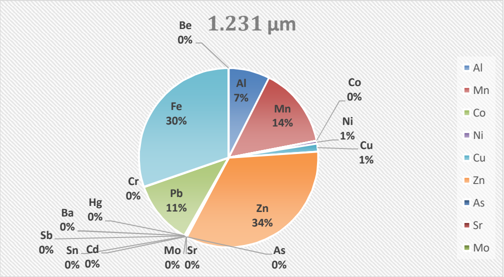

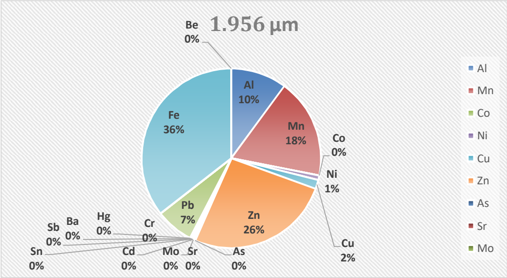

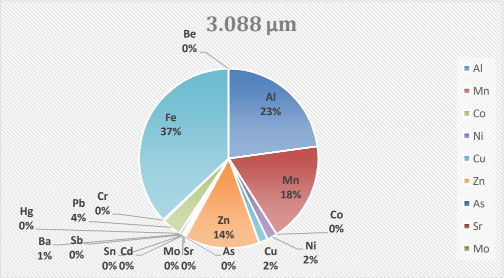

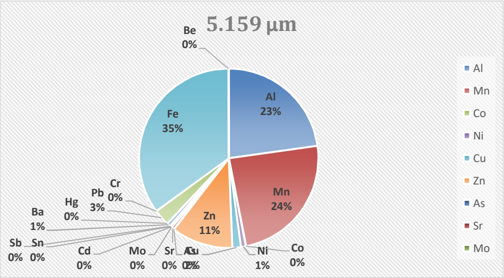

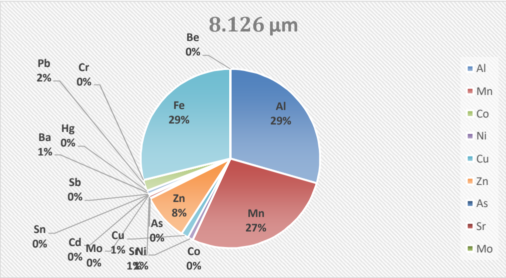

SINGLE METALLIC ELEMENTS DISTRIBUTION

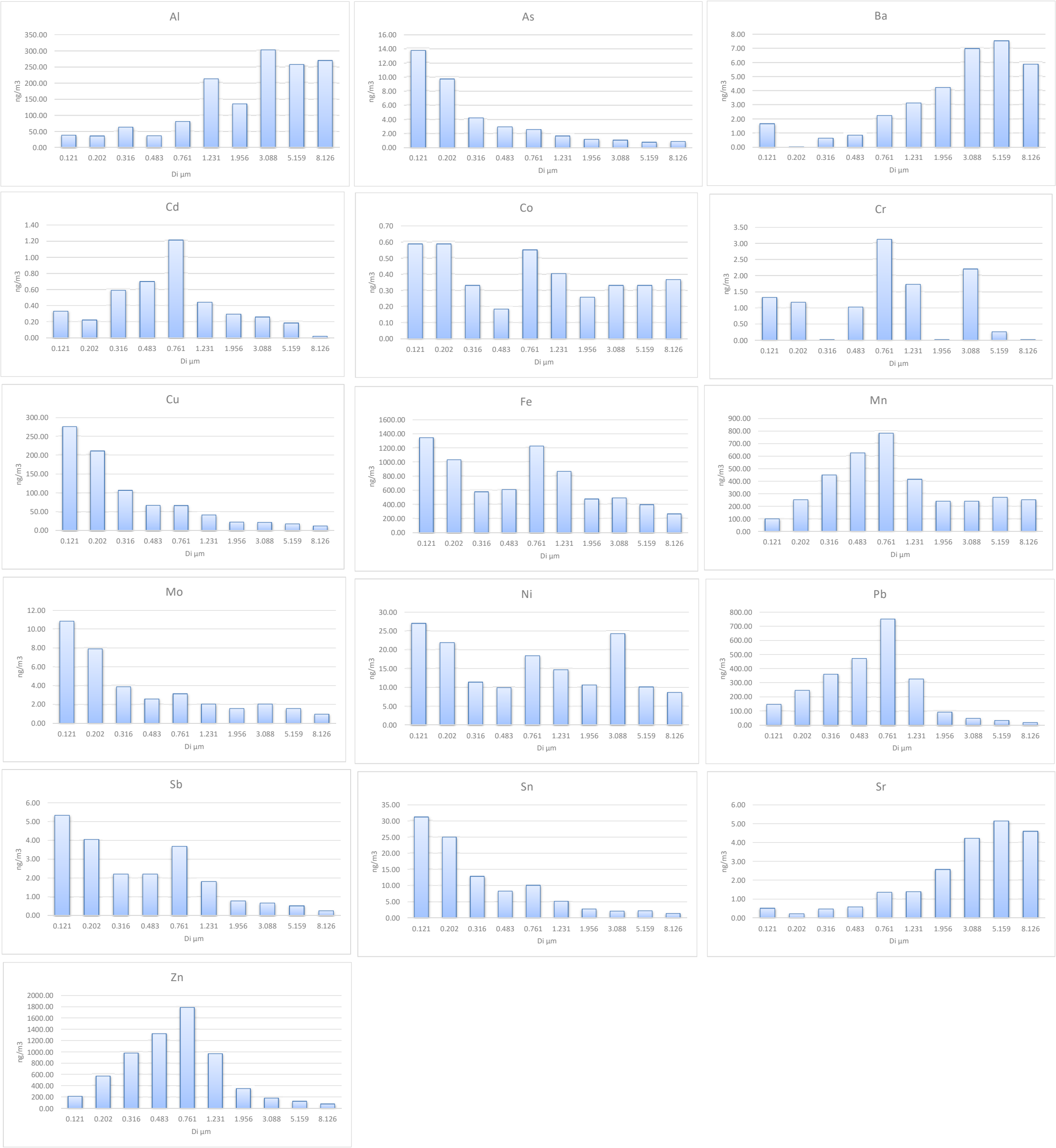

Supplement: Supplementary file 1 [file ijerph-15-01192-s001.zip › Chemical characterization of particles collected by ELPI.pdf]
